# Corrigendum to: Downregulating integrin subunit alpha 7 (ITGA7) promotes proliferation, invasion, and migration of papillary thyroid carcinoma cells through regulating epithelial-to-mesenchymal transition

**DOI:** 10.3724/abbs.2024200

**Published:** 2025-02-25

**Authors:** Yaoyao Guan, Adheesh Bhandari, Erjie Xia, Lingguo Kong, Xiaohua Zhang, Ouchen Wang

*Acta Biochim Biophys Sin* 2020, 52(2): 116–124 

https://doi.org/10.1093/abbs/gmz144

In the original version of this manuscript, an error was found in Figure 4. The correct figure is as follows. The authors apologize for the error.

**Figure:**
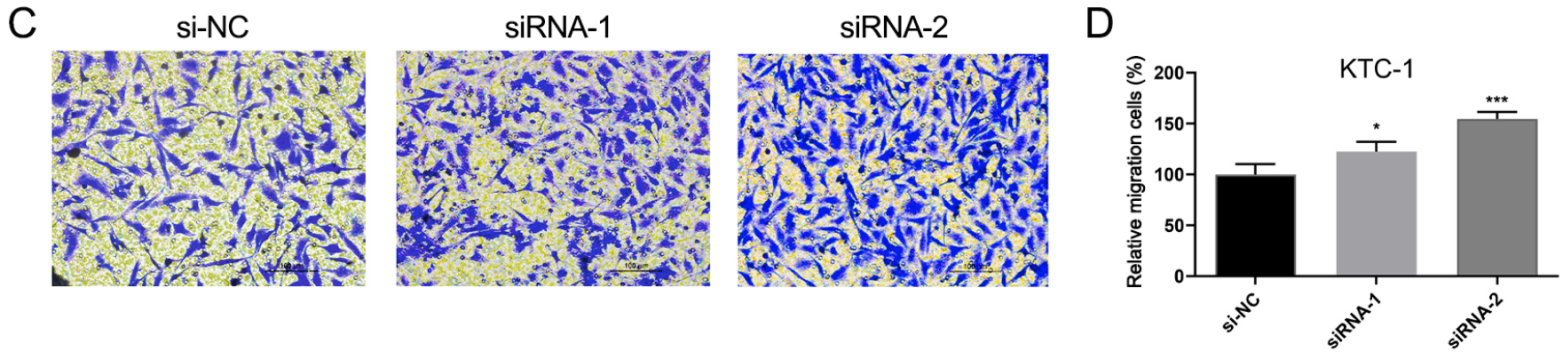
Figure 4 Downregulation of ITGA7 gene expression promotes cell migration of PTC cells (A,C) Cell migration assays in ITGA7-knockdown cells and their corresponding control cells. (B,D) Quantitative results of migration assays. The stained cells were manually counted from five randomly selected fields. *P < 0.05, **P < 0.01, ***P < 0.001 compared with the si-NC group.

